# Complete Genome Sequence of Duck Tembusu Virus Isolated from Pekin Ducks in Shanghai, China

**DOI:** 10.1128/genomeA.00308-15

**Published:** 2015-04-23

**Authors:** Yuqiang Cheng, Chuanpeng Zhang, Hengan Wang, Yaxian Yan, Chan Ding, Jianhe Sun

**Affiliations:** a School of Agriculture and Biology, Shanghai Jiao Tong University, Shanghai Key Laboratory of Veterinary Biotechnology, Key Laboratory of Urban Agriculture (South), Ministry of Agriculture, Shanghai, China; b Shanghai Veterinary Research Institute, Chinese Academy of Agricultural Sciences, Shanghai, China

## Abstract

We report here the complete genomic sequence of the duck Tembusu virus (DTMUV) SH001 strain, isolated from Pekin ducks in Shanghai, China, in 2013. The genome of SH001 is 10,990 nucleotides (nt) in length and contains a single open reading frame encoding a putative polyprotein of 3,425 amino acids.

## GENOME ANNOUNCEMENT

In 2010, a novel flavivirus emerged in eastern China and caused an extensive outbreak among egg-laying and breeder ducks (1). The pathogen caused high morbidity, up to 100%, and <5% mortality. The egg production rates dropped drastically to 10% within 5 days. Postmortem examinations showed severe ovarian hemorrhage, ovaritis, and regression (2). Up to now, several complete genomic sequences of duck Tembusu virus (TMUV) have been reported in many provinces of China (3–6), but there have been few reports about this virus isolated in Shanghai.

In October 2013, a TMUV strain was isolated from a duck farm with an outbreak of egg drop syndrome in Pekin ducks in Shanghai, China. Samples from the liver and theca folliculi from the affected ducks were used for virus isolation, and the isolated virus strain was named SH001. The animal experiments were conducted according to the Guidelines for Animal Experimentation of the Shanghai Veterinary Research Institute. Eleven pairs of overlapping PCR primers were designed using Oligo 6 (version 6.71; Wojciech Rychlik, USA), according to the three published complete genome sequences of TMUV, WFZ_2012 (GenBank accession no. KC990545), the chicken-isolated strain CK-SD-11 (GenBank accession no. JQ627862), and the goose-isolated strain GS-PT-7 (7, 8). RNA extraction was used, according to the manufacturer’s instructions, for the synthesis of viral cDNA using the Moloney murine leukemia virus (M-MLV) reverse transcriptase (Promega, USA) with random primers. Eleven overlapping fragments of TMUV cDNA were amplified using PrimeSTAR HS polymerase (TaKaRa, Dalian, China). Last, all of the amplified products were purified and cloned into the pMD19-T vector (TaKaRa) and sequenced in triplicate. The sequences were assembled using the SeqMan programs (DNAStar) to produce the final genome sequence.

Sequence analysis showed that the full genomic length of SH001 is 10,990 nucleotides (nt). The sequence contains a long open reading frame (ORF) of 10,278 nt (nt 95 to 10372), encodes a polyprotein of 3,425 amino acids (aa), and contains eight nonstructural proteins (nonstructural 1 [NS1], NS2A, NS2B, NS3, NS4A, 2K, NS4B, and NS5) and three structural proteins (capsid, PrM, and envelope). Comparative sequence analysis revealed that the nucleotide homology of SH001 ranged from 98.5% to 98.8% compared to that of the other Tembusu virus strains available in the NCBI database. Specifically, the nucleic acid (98.8%) and amino acid (99.5%) sequences of SH001 are more closely related to strain BYD-1, a duck-origin strain isolated in 2011 (4).

We report here the complete genome sequence of Tembusu virus strain SH001. The data contribute to the exploration of the molecular epidemiology and evolutionary of the Tembusu virus. Moreover, the mutation sites of different strains might reveal the mechanisms of molecular epidemiology and molecular pathogenesis.

### Nucleotide sequence accession number. 

The whole-genome sequence of duck Tembusu virus strain SH001 has been deposited in GenBank under the accession no. KP742476.
